# Non-Destructive Detection Model and Device Development for Duck Egg Freshness

**DOI:** 10.3390/foods15071211

**Published:** 2026-04-02

**Authors:** Qian Yan, Qiaohua Wang, Meihu Ma, Zhihui Zhu, Weiguo Lin, Shiwei Liu, Wei Fan

**Affiliations:** 1 College of Engineering, Huazhong Agricultural University, Wuhan 430070, China; yqyq@webmail.hzau.edu (Q.Y.); zzh@mail.hzau.edu.cn (Z.Z.); linweiguo@mail.hzau.edu.cn (W.L.); hustliusw@sina.com (S.L.); fanwei@mail.hzau.edu.cn (W.F.); 2 Ministry of Agriculture Key Laboratory of Agricultural Equipment in the Middle and Lower Reaches of the Yangtze River, Wuhan 430070, China; 3 National Research and Development Center for Egg Processing, Huazhong Agricultural University, Wuhan 430070, China; 4 College of Food Science and Technology, Huazhong Agricultural University, Wuhan 430070, China; mameihuhn@163.com

**Keywords:** Haugh Units, multi-angle transmission imaging, weighted Otsu algorithm, LAB color space clustering, poultry egg grading, image segmentation

## Abstract

To address the low accuracy of traditional freshness detection/grading and poor adaptability to different shell colors in the duck egg industry, this study developed a non-destructive detection model and an integrated device for duck egg freshness based on machine vision combined with eggshell optical property analysis. A four-sided yolk transmission imaging system was designed, and accurate yolk region segmentation was achieved via grayscale conversion, a weighted improved Otsu algorithm for whole-egg segmentation, histogram equalization enhancement, and K-means clustering in the LAB color space. A relational model between the average four-angle yolk projected area ratio and Haugh Units (HU) freshness grades was constructed, with grading thresholds determined by constrained optimization combined with the Youden index to balance food safety and grading accuracy. Experimental results showed the model achieved an overall freshness grade discrimination accuracy of 91.3%, with a sensitivity of 97.1% and specificity of 98.9% for inedible Grade B (HU < 60) duck eggs and below. An automated testing device was further developed, adopting a roller-rotating motor collaborative mechanism for automatic flipping and imaging, and equipped with a 10 W/5500 K LED cool white light source to solve the problem of poor adaptability to different shell colors. The device achieved an overall discrimination accuracy of 88.5% with a detection time of ≤5 s per egg, and its host computer can real-time output the yolk area ratio, predicted HU value, and freshness level. This study provides a high-precision and low-cost technical solution for the refined grading of the poultry egg industry.

## 1. Introduction

The poultry egg industry is a core pillar of China’s agricultural and rural economy. As a key raw material for traditional egg products (e.g., preserved eggs, salted eggs), the freshness of duck eggs directly determines the quality, stability and safety of processed products [1]. China is the world’s largest producer of duck eggs, with an annual output accounting for approximately 15% of the total poultry egg production, over 80% of which is used for processing [2]. A 2024 consumer survey showed that nearly 90% of respondents ranked “freshness” as the top consideration when purchasing poultry eggs [3]. However, duck eggs with insufficient freshness are prone to defects such as egg white coagulation, rough egg yolk, and flavor loss after processing, which not only cause raw material waste but also may trigger food safety complaints [4]. The internationally recognized Haugh Unit (HU) system classifies duck eggs into Grade AA (HU ≥ 72, suitable for high-end reprocessed eggs), Grade A (60 ≤ HU < 72, requiring adjusted processing technology), and Grade B (HU < 60, restricted processing applications) [5]. Nevertheless, traditional detection methods struggle to achieve accurate grading, hindering the transformation of the egg product processing industry towards refinement and high added value.

Currently, duck egg freshness detection mainly relies on destructive biochemical detection and manual light inspection [6]. Although destructive biochemical detection offers high precision, it requires shell breaking and sampling, failing to meet the demand for non-destructive screening of raw materials. Manual light inspection is characterized by high subjectivity and low efficiency, making it difficult to adapt to large-scale processing lines. Scholars at home and abroad have explored multiple technical pathways, forming mainstream directions such as spectral technology [7,8,9,10,11], acoustic characteristics [12], electronic nose [13], and machine vision [14,15]. Hyperspectral imaging technology achieves an accuracy of 95% in egg freshness detection by virtue of multi-band information [16], but its high equipment cost (single unit ≥ 500,000 RMB) and cumbersome data processing make it unaffordable for small and medium-sized egg product enterprises [17]. Electronic nose technology takes more than 2 min for a single detection [13], failing to meet online processing requirements. Acoustic technology is susceptible to workshop noise interference, with a detection accuracy of only 78% [12]. Machine vision technology is widely used in agricultural product quality detection due to its moderate cost and high efficiency [18,19,20], but significant structural differences between duck eggs and chicken eggs limit the direct transfer of existing chicken egg detection technologies. In addition, existing duck egg freshness detection models suffer from poor generalization ability, single shell color adaptability, and high segmentation error rates [21,22,23], which lead to low practical applicability in industrial production.

Duck eggshells are thicker, and the carotenoid content of blue-green duck eggshells reaches 12.3 ± 1.5 μg/g, resulting in poor light transmittance [21]; the egg yolk is an irregular ellipsoid with eccentric distribution, and single-angle images are prone to introducing errors in feature extraction [22]. Existing studies on duck egg freshness detection still have limitations: the regression model based on blue-green duck eggs proposed by Liu et al. has a correlation coefficient of only 0.767 [21], indicating insufficient generalization ability; the model developed by Wang et al. is only applicable to white-shell duck eggs [23], with poor adaptability to different shell colors; the reflective detection method by Ma has an error rate exceeding 8% due to occlusion issues [22]. In addition, most studies only focus on detection accuracy, lacking in-depth analysis of the mechanism underlying yolk morphological changes, and the modeling tends to rely on empirical fitting [24].

Spectral and acoustic technologies have inherent drawbacks such as high cost and poor anti-interference ability, while traditional machine vision methods fail to solve the problems of shell color adaptability and single-angle detection errors. In view of this, this study proposes the core hypothesis: by analyzing the osmosis-driven mechanism of yolk morphology during duck egg storage, combined with four-angle transmission imaging and accurate image processing, the bottlenecks of poor adaptability to different shell colors and large errors from single-angle detection can be broken through, a high-precision and universal detection model can be constructed, and the developed integrated device can meet industrial needs. Centering on this hypothesis, the following main work was carried out: (1) analyzing the evolutionary mechanism of yolk morphology, clarifying the law of water migration dominated by the osmotic pressure between egg white and yolk, and establishing a quantitative correlation between yolk projected area and HU; (2) optimizing machine vision methods, designing a four-angle transmission imaging system, and realizing accurate segmentation of duck egg yolk by combining the improved Otsu algorithm and LAB space K-means clustering; (3) constructing a discrimination model based on the multi-angle yolk projected area ratio, determining grading thresholds through constrained optimization with the Youden index to balance detection accuracy and food safety; (4) developing an integrated device, integrating automatic flipping and imaging with a GUI host computer to achieve detection automation and real-time result output, and verifying the stability of the device.

## 2. Materials and Methods

### 2.1. Experimental Materials

A total of 500 fresh blue-green and white-shell Maya duck eggs produced by healthy domestic ducks from a large-scale farm in Hubei Province were selected for the experiment. The eggs were transported to the laboratory within 24 h after harvesting, and damaged, contaminated, and malformed samples were screened out, leaving 450 eggs for the test. Samples were stored in a constant temperature and humidity incubator (Minto Technology, Chengdu, China) to simulate industrial shelf conditions (temperature: 26 °C, relative humidity: 60%), which was consistent with the actual circulation and storage environment of duck eggs [21]. From the 450 eggs, 20 were randomly selected as the control group to track changes in yolk morphology. The test period was 36 days, with sampling conducted on days 0, 4, 8, 12, 16, 20, 24, 28, 32, and 36 of storage. Forty eggs were randomly selected each time, and the remaining 30 served as backup samples to replace accidentally damaged or contaminated samples during the experiment, ensuring data continuity.

### 2.2. Experimental Setup

During duck egg storage, there is an osmotic pressure difference between the egg white and yolk (approximately 280 mOsm/kg for egg white and 320 mOsm/kg for yolk) [25]. Moisture in the egg white continuously permeates into the yolk, while salts in the yolk diffuse into the egg white, leading to decreased elasticity of the yolk membrane and gradual increase in yolk volume until rupture and dispersion [26]. Specifically, yolk volume change is negatively correlated with freshness. Two-dimensional projected area can effectively characterize three-dimensional volume changes; however, due to the yolk’s irregular ellipsoidal shape and eccentric distribution, single-angle projected area introduces significant errors. Multi-angle imaging can substantially reduce such systematic errors; thus, a four-sided (0°, 90°, 180°, 270°) imaging scheme was adopted in this study.

**Figure 1:**
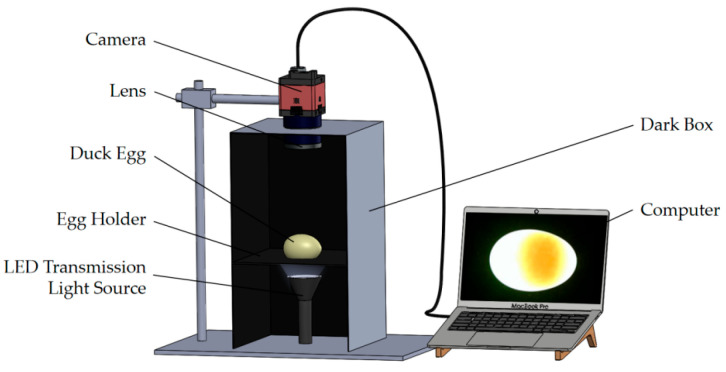
Yolk image acquisition device.

A duck egg yolk image acquisition device was constructed (Figure 1), consisting of a cuboid closed dark box with black-painted inner walls to avoid stray light interference. A Zhongwei Aoke wide-angle camera (Shenzhen, China) with a 4.3 mm focal length and 1920 × 1080 resolution was mounted 15 cm directly above the egg holder. The center of the egg holder featured an elliptical hole (major axis: 33 mm, minor axis: 25 mm) to ensure the duck egg’s major axis was parallel to the holder’s plane. An LED transmission light source (illumination area: 20 cm^2^, Oren Trading, Zhongshan, China) was installed beneath the egg holder, with the light exit tube aligned vertically. When the light source was activated, light beams penetrated the eggshell into the duck egg, resulting in differences in illumination intensity and color between the yolk and egg white regions, thereby visualizing the position and morphology of the yolk.

An L9 (3^3^) orthogonal experiment was designed to optimize the light source parameters (power: 5 W/10 W/20 W; color temperature: 5000 K/5500 K/6000 K), with the yolk segmentation Intersection over Union (IoU) as the evaluation metric. All experimental data analysis was performed using Matlab R2025a (MathWorks, Natick, MA, USA), including analysis of variance (ANOVA), linear regression, and K-means clustering. ANOVA was used to test the statistical significance of light source parameters on IoU (*p* < 0.05).

**Figure 2:**
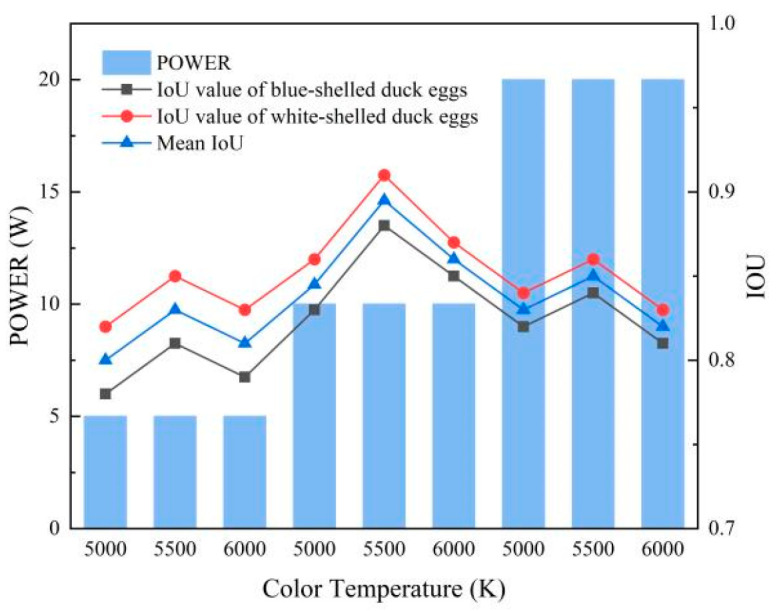
Orthogonal test results of light source parameters.

**Figure 3:**
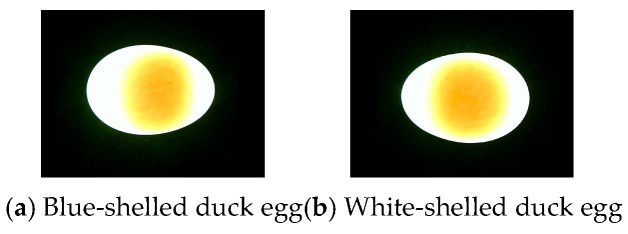
Typical duck egg yolk image under transmission light.

**Figure 4:**
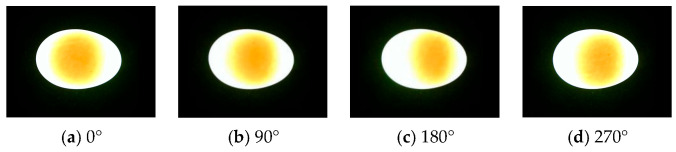
Transmitted light images of the same duck egg from different sides.

Figure 2 shows the orthogonal test results of light source parameters, and the optimal light source configuration was determined to be a 10 W, 5500 K neutral white LED. Under these light source parameters, the difference in light transmittance between blue-green and white-shell duck eggs is relatively minimal, while the grayscale contrast between the yolk and egg white is maximized, meeting the requirements for accurate segmentation. Figure 3 shows typical images of blue-green and white-shell duck eggs under this transmission light source, and Figure 4 shows the transmission light images of the same duck egg captured from four different angles.

As shown in Figure 3, under this light source, there is no significant difference in the image quality of blue-shelled and white-shelled duck eggs, with the yolk region’s shape and size clearly visible to the naked eye. Therefore, blue-shelled and white-shelled duck eggs were mixed to build a freshness discrimination model. It can be observed from Figure 4 that there are significant differences in the yolk morphology and size among transmission light images obtained from different sides, indicating that single-angle image acquisition will affect the reliability of detection results. Thus, transmission light images from four angles were collected and averaged to objectively reflect the actual size of the duck egg yolk.

### 2.3. Experimental Methods

First, four-angle transmission images of each duck egg were collected via the designed device. Subsequently, the eggs were broken to determine HU values within 1 min for confirming freshness grades, and a relational model between characteristic parameters and freshness grades was established. The 400 samples were divided into a modeling set (240 samples) and a validation set (160 samples) at a ratio of 3:2, with 5-fold cross-validation used for model training to avoid overfitting. A single-angle (0°) imaging model was also established for comparative analysis to quantify the advantage of multi-angle imaging.

#### Haugh Unit (HU) Measurement Protocol

HU values were measured following the standard method used by Raymond Haugh [5] to minimize experimental errors, with the detailed protocol as follows:

1. Instruments: Electronic balance (0.01 g precision, FA2004, Jingke Instrument, Shanghai, China) for egg weight (W) measurement; digital vernier caliper (0.01 mm precision, MDH-200, Miaozhi Instrument, Shenzhen, China) for albumen height (H) measurement (measured at three points around the yolk, and the average value was adopted).

2. Calibration: All instruments were calibrated daily with national metrology-certified standard weights and gauge blocks.

3. HU Formula:

(1) $$HU=100\times lg\left(H+7.57-1.7\times W^{0.37}\right)$$

4. Operator Variability: Two trained operators each performed three replicate measurements; the average value was adopted, with a relative standard deviation (RSD) < 5%.

5. Timing Control: HU measurements were completed within 1 min after egg cracking to avoid albumen dehydration and shrinkage.

### 2.4. Image Processing and Feature Extraction

**Figure 5:**
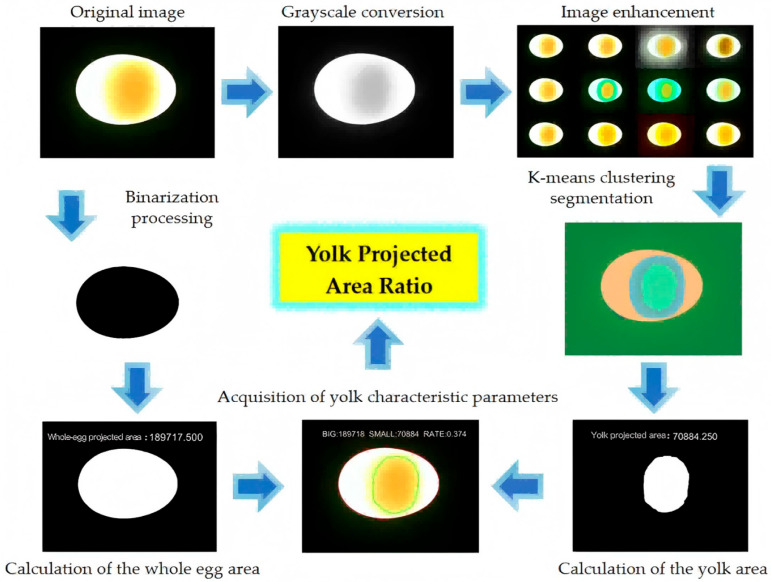
Yolk image processing flowchart.

Matlab R2025a software was used for image processing [27,28] to accurately segment the yolk region and extract its morphological characteristic parameters. The acquired transmission images of duck eggs were processed through a series of algorithms, including grayscale conversion, whole egg segmentation, image enhancement [29], yolk segmentation, and calculation of the area ratio of yolk to whole egg, to obtain the target yolk characteristic parameters. The flowchart of the image processing procedure is shown in Figure 5.

#### 2.4.1. Whole-Egg Segmentation via Weighted Improved Otsu Algorithm

To extract geometric features of yolk transmission images, the original RGB images were first converted to grayscale images to reduce image information volume. Significant differences exist in grayscale values between the whole egg region and the surrounding background; thus, the weighted improved Otsu algorithm [30] was adopted for whole egg segmentation. This algorithm introduces a pixel weight coefficient $\omega =\left(\sigma_{b}/\sigma_{t}\right)\times \left(N_{b}/N_{t}\right)$ (where $\sigma_{b}$ = between-class variance, $\sigma_{t}$ = total variance, $N_{b}/N_{t}$ = background/target pixel ratio) based on the traditional Otsu algorithm, which adaptively adjusts the segmentation threshold according to grayscale intensity differences, avoiding edge loss or background misjudgment caused by single-threshold segmentation.

To smooth the contour of the whole egg in the image and weaken narrow parts, morphological processing was performed sequentially, including opening operation, closing operation, and removal of small-area white regions in the binary image. Functions imopen, imclose, and bwareaopen were called in turn in Matlab. Finally, the function bwarea was used to calculate the number of white pixels in the image, which corresponds to the pixel area of the whole egg projection.

#### 2.4.2. Yolk Segmentation via LAB Space K-Means Clustering

Image enhancement was conducted to improve the recognizability of the yolk boundary. Functions imadjust, histeq, and adapthisteq were called to perform grayscale adjustment, histogram equalization, and adaptive histogram equalization for contrast enhancement on the original RGB images [31], respectively. K-means clustering [32] was used to classify colors in the LAB color space for yolk segmentation, with the following parameter settings:

1. Initialization: K-means++ algorithm to avoid random cluster center bias;

2. Stopping criteria: Iteration number ≥ 100 or cluster center change < 1 × 10^−6^;

3. Cluster number: K = 3 (determined via the Elbow Method, with a significant inflection point in the sum of squared errors (SSE) at K = 3).

**Figure 6:**
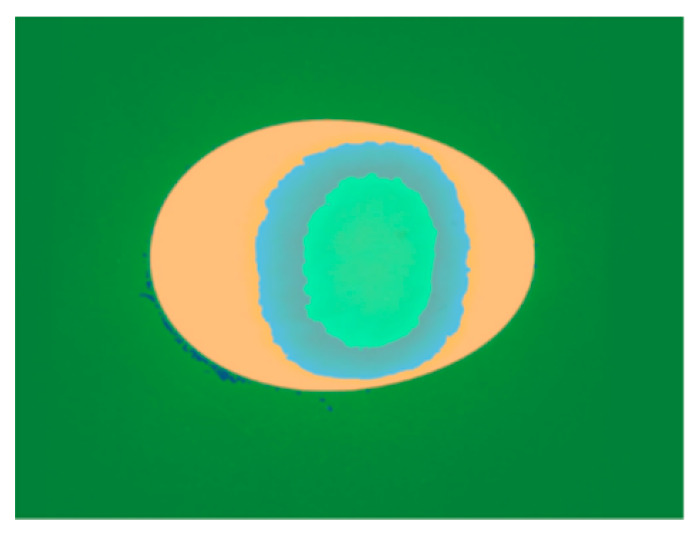
K-means clustering segmentation of yolk image.

The enhanced yolk region was segmented using the labeloverlay function, and the segmentation result is shown in Figure 6. The orange region represents the egg white, the blue region corresponds to the ghosting of the yolk projection under transmission light, and the central green region is the target yolk region.

#### 2.4.3. Characteristic Parameter Extraction

**Figure 7:**
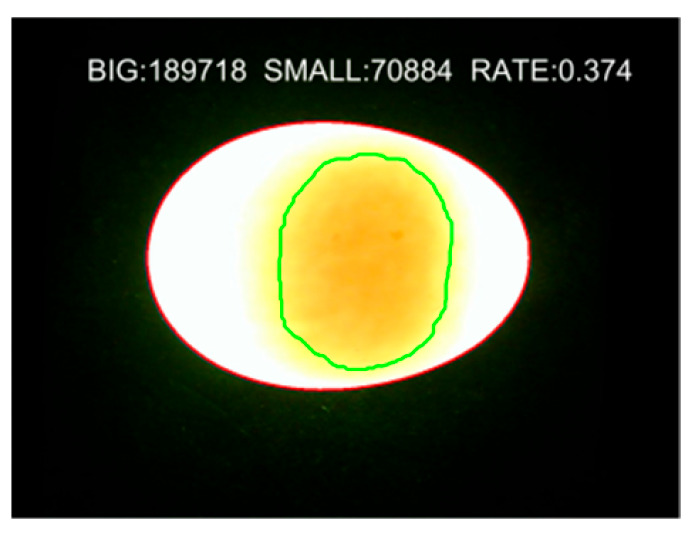
Calculation and acquisition of duck yolk characteristic parameters (BIG: Whole-egg projected pixel area; SMALL: Yolk projected pixel area; RATE: Yolk projected area ratio).

With the extension of storage time, the yolk volume continues to increase, and its projected area under the transmission light source also increases accordingly. Statistics were collected on the total projected pixel area of the whole duck egg and the pixel area of the target yolk region. The ratio of the two—the Yolk Projected Area Ratio—serves as the yolk characteristic parameter for determining the duck egg freshness grade. This parameter can effectively eliminate the impact of individual differences among duck eggs on freshness discrimination results, while avoiding the complexity and large computation load of directly calculating the actual size of the duck egg yolk region. The extraction results are shown in Figure 7.

The Yolk Projected Area Ratio was calculated, and the average value of four angles was defined as Sn; the formulas are as follows:

(2) $$S=\frac{S_{SMALL}}{S_{BIG}}\times 100\%$$

(3) $$S_{n}=\frac{S_{1}+S_{2}+S_{3}+S_{4}}{4}$$

where *S_SMALL_* denotes the yolk projected area; *S_BIG_* denotes the whole-egg projected area, and *S*_1–4_ represent the yolk projected area ratios obtained from the four different angles (0°, 90°, 180°, 270°), respectively.

#### 2.4.4. IoU Evaluation Protocol

The ground-truth masks for yolk segmentation were manually annotated by two trained professionals based on the actual yolk contour observed after egg cracking, with a Kappa coefficient of 0.92 (indicating high annotation consistency).

A total of 60 images (30 blue-green shell, 30 white shell) were randomly selected for IoU evaluation to quantify the segmentation performance across shell colors.

## 3. Results and Analysis

### 3.1. Selection of the Nominal Power of the LED Light in the Detection System

The L9 (3^3^) orthogonal experiment results showed that the light source power and color temperature had significant effects on the yolk segmentation IoU (*p* < 0.05), while their interaction was not significant (*p* > 0.05). The 10 W/5500 K LED light source achieved the highest mean IoU (0.92), with the quantitative segmentation performance across shell colors as follows: blue-green shell (IoU: 0.91 ± 0.03), white shell (IoU: 0.93 ± 0.02). This configuration minimized the light transmittance difference between blue-green and white-shell duck eggs and maximized the grayscale contrast between the yolk and egg white, which was selected as the optimal light source parameter for subsequent experiments.

### 3.2. Data Analysis of the Control Group

**Figure 8:**
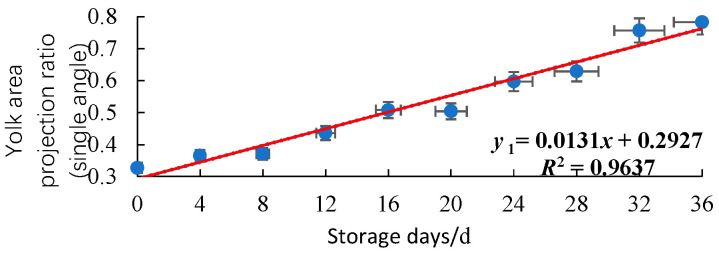
Relationship plot between the yolk projected area ratio at 0° single angle and storage days (n = 20).

**Figure 9:**
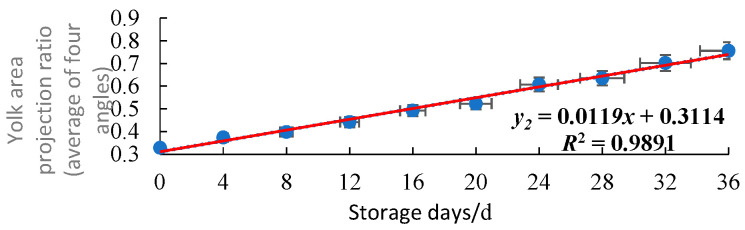
Relationship plot between the yolk projected area ratio at four angles (average) and storage days (n = 20).

For the 20 duck eggs in the control group, images were collected every 4 days to plot the relationship plot between the yolk projected area ratio and the storage days, as shown in Figure 8 and Figure 9.

It can be seen from the above figures that with the extension of storage days, the freshness of duck eggs gradually decreases, and the yolk projected area ratio increases gradually in a linear trend. The coefficient of determination R^2^ of the regression equation between the average value of four angles and storage days is 0.9891, while that between the 0° single-angle value and storage days is 0.9637. This indicates that taking the average of four angles can effectively reduce the error caused by the eccentric distribution of the yolk.

### 3.3. Establishment and Validation of the Discriminant Model

#### 3.3.1. Threshold Determination via Constrained Optimization and Youden Index

Statistical analysis was performed on the average value of the yolk projected area ratio from four angles of duck eggs in the experimental group and the freshness grades determined by HU values. The distribution of the average yolk projected area ratio of duck eggs at different freshness grades was obtained, as shown in Table 1.

It can be seen from Table 1 that the average yolk projected area ratio of Grade AA duck eggs is the smallest, followed by Grade A duck eggs, and that of Grade B and below duck eggs is the largest. There is a certain degree of overlap in the distribution intervals of adjacent freshness grades, which is mainly caused by the non-one-to-one correspondence between HU and yolk characteristic parameters, individual differences in duck eggs, and minor image segmentation errors.

To ensure food safety, constrained optimization was used to prioritize the detection accuracy of inedible Grade B and below duck eggs, combined with the Youden index (J = Sensitivity + Specificity − 1) to determine the optimal grading thresholds. The maximum Youden index (J = 0.96) for Grade B and below duck eggs was achieved at S_n_ = 54.3%, and the threshold for Grade AA was determined at S_n_ = 38.2% (Youden index J = 0.91). Finally, the grading thresholds were determined as follows:

• Grade AA: S_n_ ≤ 38.2%
• Grade A: 38.2% < S_n_ < 54.3%
• Grade B and below: S_n_ ≥ 54.3%

#### 3.3.2. Model Validation and Classification Performance

**Figure 10:**
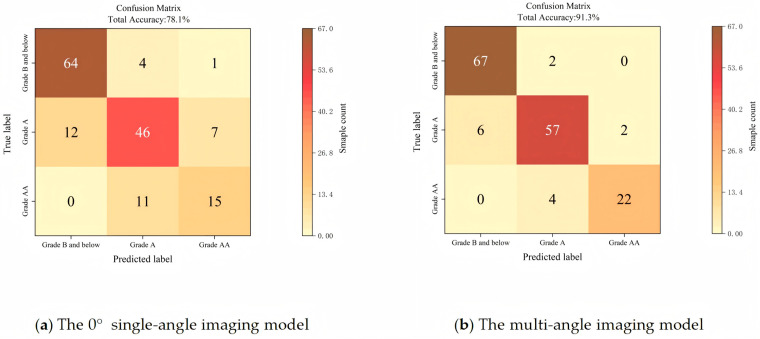
The confusion matrix of the model (validation set, n = 160).

The validation set (160 samples) was used to test the proposed model, and the confusion matrix of the model is shown in Figure 10. For inedible Grade B and below duck eggs, the model achieved a sensitivity of 97.1% and specificity of 98.9%, which effectively minimized the misclassification of inferior duck eggs into the edible grade.

The sample sizes of different grades (26, 65, 69) were consistent with the natural distribution of duck egg freshness grades during storage (fewer high-freshness Grade AA eggs with extended storage time). The weighted accuracy of the model (calculated based on grade proportion) was 91.5%, which was consistent with the overall accuracy (chi-square test, χ^2^ = 1.23, *p* > 0.05), indicating that the sample size imbalance did not increase the experimental error.

The 0° single-angle imaging model achieved an overall classification accuracy of only 78.1%, with a recall of 89.5% for Grade B and below duck eggs. The multi-angle model significantly improved the classification performance, verifying the advantage of four-angle imaging in reducing the error caused by yolk eccentric distribution.

#### 3.3.3. HU Linear Regression Model

A univariate linear regression model was directly fitted to the measured HU values and four-angle average S_n_ (modeling set: 240 samples; validation set: 160 samples). The regression model was established as follows:

(4) $$HU=-74.14\times S_{n}+101.29$$

where the values after ± represent the standard error of the regression coefficients. The coefficient of determination R^2^ between the predicted values of the model and the measured values is 0.978, and the Mean Absolute Error (MAE) is 1.86. Residual analysis showed that the residuals followed a normal distribution (Shapiro–Wilk test, W = 0.98, *p* > 0.05), which verified the linearity of the relationship between Sn and HU. Nonlinear regression models (e.g., quadratic, exponential) were also fitted for comparison, and the results showed that the linear model achieved the highest R^2^ and the lowest MAE; thus the linear model was selected for HU prediction.

### 3.4. Performance of the Testing Device and Software

**Figure 11:**
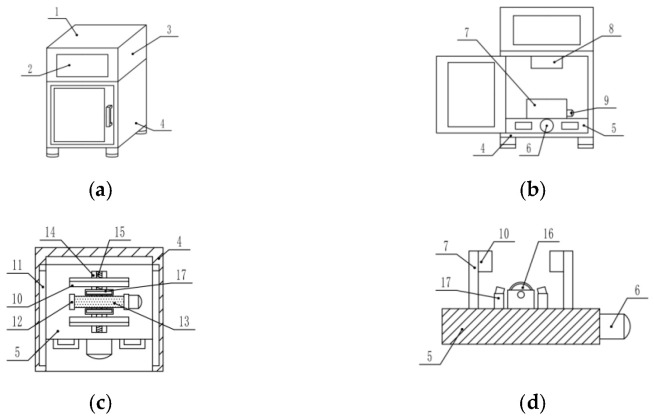
Structural schematic diagram of the integrated testing device. 1. Control box. 2. Touchscreen display. 3. Industrial computer. 4. Detection device body. 5. Detection platform. 6. Bidirectional motor. 7. First baffle. 8. Camera. 9. Rotary motor. 10. Cushion pad. 11. Slideway. 12. Second baffle. 13. Anti-slip pad. 14. Moving slot. 15. Bidirectional screw. 16. Roller. 17. Transmission light source. (**a**) Schematic diagram of the device’s external structure. (**b**) Front view of the internal structure. (**c**) Top view cross-section diagram. (**d**) Side view schematic of the detection platform.

Based on the above research methods, a non-destructive integrated testing device for duck egg freshness based on multi-angle yolk characteristics was designed. The overall structure is divided into two main parts: the processing module and the detection device, as shown in Figure 11.

The main body of the integrated testing device consists of a test bench, a roller, baffles, a motor, a transmission light source, a camera, and other components. The roller-rotating motor collaborative mechanism can fix duck eggs of different sizes and drive them to rotate stably around their own center point, realizing automatic collection of four-angle transmission images with a detection time per egg ≤ 5 s. Buffer pads are installed on the inner sides of the baffle to avoid egg damage caused by collision.

**Figure 12:**
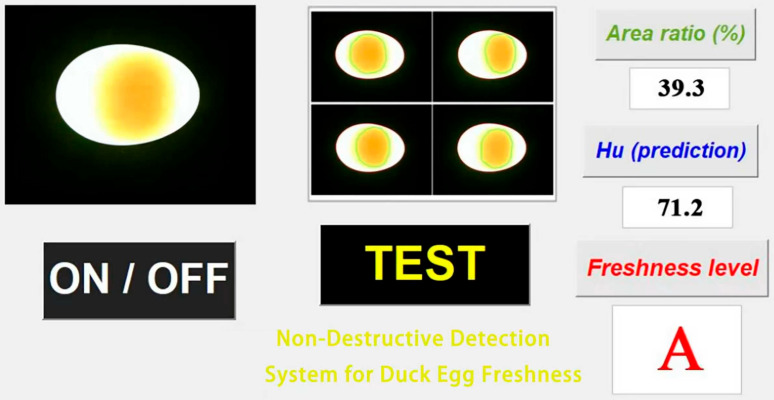
Host computer display of determination results.

The software interface was developed using the Graphical User Interface (GUI) module of Matlab [33]. The image display frame shows the real-time picture monitored by the camera and the yolk region segmentation effects at four angles. The “ON/OFF” button controls the opening and closing of the visual inspection system, and the “TEST” button initiates the detection. The text display frame real-time outputs the yolk area ratio, predicted HU value (based on Equation (1)), and freshness level. Figure 12 shows the host computer result display interface.

Another batch of 200 duck eggs with different storage periods and freshness levels was selected for the testing experiment of the device, and the results are shown in Table 2.

The overall accuracy of the device (88.5%) was slightly lower than that of the model (91.3%), and the chi-square test showed no statistically significant difference between the two results (χ^2^ = 1.56, *p* > 0.05). The minor accuracy reduction was mainly caused by tiny vibrations in the industrial environment and slight deviations in image acquisition during automatic rotation. The device achieved a recall of 96.5% for Grade B and below duck eggs, with only 3 samples misclassified as Grade A, effectively reducing the risk of inferior raw materials entering the processing link.

The sample sizes in Table 2 (31, 83, 86) were consistent with the natural industrial distribution of duck egg freshness grades. The weighted accuracy of the device was 90.2%, which was close to the overall accuracy (chi-square test, χ^2^ = 0.98, *p* > 0.05), indicating no experimental error caused by sample size imbalance. The device showed good adaptability to both blue-green and white-shell duck eggs, with a detection accuracy of 87.9% for blue-green shell and 89.1% for white shell.

### 3.5. Limitations of the Proposed Method

This study has achieved phased results in non-destructive detection of duck egg freshness, but there are still several major limitations in practical application:

1. Sample and storage condition limitations: The experimental samples only cover Maya duck eggs from a single farm in Hubei Province, without involving other mainstream duck breeds (e.g., Shaoxing duck, Jinding duck). The model was only verified under the storage condition of 26 °C/60% RH, and its adaptability to other temperature and humidity conditions needs to be further tested.

2. Interfering factors in industrial production: Common interfering factors in actual production (e.g., severe eggshell surface contamination, microcracks) were not incorporated in the experiment. Severe contamination will block light transmission, and microcracks will cause light refraction, both of which may lead to increased detection errors.

3. Detection speed limitation: The device’s detection speed (≤5 s per egg) is currently suitable for medium-speed industrial processing lines (≤720 eggs/h), and the mechanical structure needs to be optimized to adapt to high-speed lines (>1000 eggs/h).

4. Segmentation error for extreme samples: For duck eggs with severe yolk expansion (near rupture), the K-means clustering algorithm has a slight segmentation error (IoU < 0.85), which needs to be improved by combining deep learning segmentation models.

## 4. Conclusions

This study verified the core research hypothesis that four-angle transmission imaging and precise image processing can effectively break through the bottlenecks of poor shell color adaptability and large single-angle detection errors in duck egg freshness detection, and the developed model and integrated device can meet the industrial needs of refined grading.

The osmosis-driven water migration between egg white and yolk was confirmed as the main mechanism of yolk volume increase during duck egg storage, and a quantitative linear correlation between the four-angle average yolk projected area ratio and HU was established ($HU=-74.14\times S_{n}+101.29$, R^2^ = 0.978). The weighted improved Otsu algorithm and LAB space K-means clustering (K-means++ initialization, K = 3) achieved high-accuracy yolk segmentation for both blue-green and white-shell duck eggs (IoU: 0.91 ± 0.03 and 0.93 ± 0.02, respectively). The freshness discrimination model with thresholds determined by constrained optimization and the Youden index achieved an overall accuracy of 91.3%, with a sensitivity of 97.1% for Grade B and below duck eggs, effectively balancing detection accuracy and food safety.

The developed integrated testing device adopted a roller-rotating motor collaborative mechanism to realize automatic four-angle imaging (≤5 s per egg), and the 10 W/5500 K LED light source solved the problem of poor adaptability to different shell colors. The device achieved an overall detection accuracy of 88.5%, with real-time output of the yolk area ratio, predicted HU value, and freshness level, providing a high-precision and low-cost technical solution for the non-destructive detection of duck egg freshness.

Future research will collect duck egg samples from multiple producing areas and breeds nationwide; optimize the model parameters through transfer learning to improve adaptability to complex storage conditions and industrial interfering factors; explore multi-modal information fusion of near-infrared spectroscopy, acoustic features and machine vision to further improve the discrimination accuracy of extreme samples; and construct an integrated detection-grading-traceability system, linking the testing device with automated sorting robotic arms and blockchain traceability platforms in order to promote the transformation of the poultry egg industry from extensive processing to precise value-added processing.

## Figures and Tables

**Table 1:** Distribution table of average projected area ratio of duck egg yolk at four angles (n = 240).

Average Yolk Area Projection Ratio S_n_/%	Grade AA (HU ≥ 72)		Grade A (60 ≤ HU < 72)		Grade B and Below (HU < 60)	
	Proportion	Percentage	Proportion	Percentage	Proportion	Percentage
31.1 ≤ S ≤ 38.2	36/41	87.8%	3/92	3.3%	0/107	0
38.2 < S ≤ 43.6	5/41	12.2%	13/92	14.1%	0/107	0
43.6 < S < 49.7	0/41	0	38/92	41.3%	0/107	0
49.7 ≤ S < 54.3	0/41	0	28/92	30.4%	2/107	1.9%
54.3 ≤ S ≤ 78.2	0/41	0	10/92	10.9%	105/107	98.1%

**Table 2:** Discrimination results of the non-destructive testing device for duck egg freshness (n = 200).

Freshness Level Determined by Breaking Test	Actual Quantity	Detection Device Measured Result			Accuracy	Overall Accuracy
		Grade AA	Grade A	Grade B and Below		
Grade AA	31	25	6	0	80.6%	88.5%
Grade A	83	4	69	10	83.1%	
Grade B and below	86	0	3	83	96.5%	

## Data Availability

The original contributions presented in the study are included in the article, further inquiries can be directed to the corresponding author.

## References

[1] Ahmed W., Hossainy S.J., Khaliduzzaman A., Emmert J.L., Kamruzzaman M. (2023). Non-destructive optical sensing technologies for advancing the egg industry toward Industry 4.0: A review. Compr. Rev. Food Sci. Food Saf..

[2] Ma M.H., Qiu N., Huang Q., Jin Y., Jin G. (2015). Development status and characteristics of China’s egg processing industry. Agric. Eng. Technol..

[3] Cui H.J. (2025). Egg consumption continues to improve, egg prices stop falling and rise slightly. Grain and Oil Market News.

[4] Tian Y.N., Chen Y.Z., Wang Q.H. (2022). Effect of the freshness of raw eggs on the quality of preserved eggs during the curing period. Food Sci..

[5] Xu M., Liu Z.G., Huang Z., Jiang D., Xie G., Wang L., Tang R., Ding Y., Liu B., Cao Y. (2021). Research Progress on Determination Indicators and Methods of Egg Quality. Poult. Sci..

[6] Zhang J.W., Dong F.L., Wang C., Zhou X. (2025). Research progress on egg freshness non-destructive testing. China Food Saf. Mag..

[7] Wang Q.H., Zhou K., Wu L.L., Wang C.Y. (2016). Egg freshness detection based on hyper-spectra. Spectrosc. Spectr. Anal..

[8] Liu Y., Jin S., Alimu A., Jiang L., Jin H. (2024). Nondestructive detection of egg freshness based on a decision-level fusion method using hyperspectral imaging technology. J. Food Meas. Charact..

[9] Fu D.D. (2020). Research on Spectral Non-Destructive Detection Method and Device Development for Protein Content and Quality of Eggs During Storage. Ph.D. Thesis.

[10] Yao K., Sun J., Zhang B., Du X., Chen C. (2024). On-line monitoring of egg freshness using a portable NIR spectrometer combined with deep learning algorithm. Infrared Phys. Technol..

[11] Kim S. (2022). Measurement of brown eggs freshness using non-destructive UV/VIS spectroscopy and viscosity of egg white. J. Korean Soc. Food Storage Distrib..

[12] Wang S.C., Wei X.B. (2009). Correlation between egg impact response characteristics and its freshness. J. Huazhong Agric. Univ..

[13] Li J.T., Wang J., Li Y., Wei Y. (2017). Detection of egg freshness using electronic nose. Mod. Food Sci. Technol..

[14] Wang Q.H., Wang C.Y., Ma M.H. (2017). Non-Destructive Detection of Duck Egg Freshness Based on Machine Vision. J. Chin. Inst. Food Sci. Technol..

[15] Sun L., Yuan L.-M., Cai J.-R., Lin H., Zhao J.-W. (2014). Egg Freshness on-Line Estimation Using Machine Vision and Dynamic Weighing. Food Anal. Methods.

[16] Yao K.S. (2023). Research on Non-Destructive Detection of Egg Quality Based on Hyperspectral Imaging Technology. Ph.D. Thesis.

[17] Li S.B., Zhang B.Y., Lu J.S., Kong B., Liu H., Chen Q. (2025). Research on the application of hyperspectral imaging technology in food quality control and safety detection. Sci. Technol. Food Ind..

[18] Shi W., Qi H., Liu Z. (2025). HALCON Machine Vision Technology in Automated Inspection Study. Int. Core J. Eng..

[19] Roth H.P., Olmos G.M.A., Olteanu A., Böschen S. (2024). Making Media Futures: Machine Visions and Technological Imaginations.

[20] Zeuch N. (2014). Understanding and Applying Machine Vision.

[21] Liu J.Y., Wang Q.H., Xiong L.R., Chen H., Yu Y., Wen Y. (2002). Experimental study on freshness model of duck egg. J. Huazhong Agric. Univ..

[22] Ma L. (2018). Research on Non-Destructive Detection of Double-Yolk, Crack and Freshness of Duck Eggs Based on Computer Vision. Ph.D. Thesis.

[23] Wang C.Y. (2016). Non-Destructive Detection of Surface Dirt and Freshness of Duck Eggs. Master’s Thesis.

[24] Tian H.Y., Sun X.W., Lu Y.H., Bi T., Wei K., Hu M., Wang J. (2025). Establishment and optimization of rapid detection method for crude fat content in fresh eggs and egg yolks. Food Sci. Technol..

[25] Huang M., Mao Y., Li H., Yang H. (2021). Kappa-carrageenan enhances the gelation and structural changes of egg yolk via electrostatic interactions with yolk protein. Food Chem..

[26] Wang Y.J. (2025). Comparison and optimization analysis of rapid detection methods for egg freshness. China Food Ind..

[27] Baez-Lopez D. (2011). MATLAB with Applications to Engineering, Physics and Finance.

[28] Davis A.T. (2011). MATLAB Primer.

[29] Rajput S.S., Khan N.U., Singh A.K., Arya K.V. (2023). Digital Image Enhancement and Reconstruction.

[30] Xia M., He L., Gu Y., Liu X., Murengami B.G., Janneh L.L., Li R., Long Y., Grecheneva A., Fu L. (2025). Tassel counting of individual ridge from UAV RGB imagery based on YOLOv8m with deep SORT and double-step Otsu thresholding algorithm by filtering abnormal IDs for maize breeding. Comput. Electron. Agric..

[31] Zhou Z.M., Li K. (2025). Research on image enhancement algorithm based on histogram equalization. Pract. Electron..

[32] Wang H.F. (2023). Deep Neural Networks and Clustering Algorithms and Their Applications in Image Classification. Master’s Thesis.

[33] Song K.C., Kim R.J. (2025). Engineering Mathematics with MATLAB.

